# Ten years of a geriatric oncology service at a public university cancer centre in Brazil

**DOI:** 10.3332/ecancer.2023.1596

**Published:** 2023-08-31

**Authors:** Theodora Karnakis, Ana L Kanaji, Isabella F Gattás-Vernaglia, Izabela O Adriazola, Paola T Ramos, Maria Eduarda P L S Lima, Olga L S Almeida, Wilson Jacob-Filho, Eduardo Ferriolli

**Affiliations:** 1 Department of Internal Medicine, Division of Geriatrics, University of São Paulo Medical School, São Paulo, Brazil; 2 Department of Internal Medicine, Division of Geriatrics, Sao Paulo Cancer Institute, University of Sao Paulo Medical School, São Paulo, Brazil; 3 Clinical Board of the Clinics Hospital of the University of São Paulo Medical School, Sao Paulo, Brazil; 4 Laboratory of Medical Investigation in Aging (LIM 66) of São Paulo Medical School, Sao Paulo, Brazil

**Keywords:** geriatric oncology, Latin America, health service research, geriatric assessment, health care, aging

## Abstract

The implementation of a geriatric oncology service is challenging in both high-income and low-and-middle-income countries. The Octavio Frias de Oliveira Institute of Cancer of Sao Paulo (ICESP) is a tertiary healthcare complex of the Clinics Hospital of the University of Sao Paulo Medical School and is considered a model of excellence in oncology in Latin America. The objective of this manuscript is to describe 10 years of the geriatric oncology service at ICESP and the challenges for its implementation. We performed a narrative description of the ICESP’s geriatric oncology service and a general retrospective descriptive analysis of data collected from routine structured medical records of patients referred to the service from 2011 to 2021. This article highlights the different settings in which the service operates (outpatient, pre-operative and hospital follow-up). In this period, 1,700 patients were assessed for preoperative evaluation (median age 83.9, SD 4.95), 468 patients were evaluated for therapeutic decision (median age 79.4, SD 7.38), 968 in general geriatric oncology care outpatient clinics from 2012 to 2021 (median age 78.7, SD 7.91) and 1,391 inpatient evaluations. In the past 10 years, our geriatric oncology team has grown exponentially and changed its characteristics in order to adjust them to the hospital demands, raising awareness among the oncology teams about the benefit of using geriatric assessment and promoting multidisciplinary discussions.

## Background

Brazil is the largest country in South America, with a population of approximately 214 million inhabitants, and has a notable diversity in human development rates and access to healthcare. Like most Latin American and other low-and middle-income countries (LMIC), Brazil has an accelerated aging process. Currently, older adults account for 14.7% of the total population and are estimated to represent 18.6% of the population by 2030 and 33.7% by 2060 [1].

Population aging directly affects the incidence of chronic degenerative diseases, of which cancer is one of the most challenging owing to its complexity in terms of diagnosis and treatment. Similar to what happened decades ago in high-income countries (HICs), a growing number of cancer diagnoses is also a reality in Brazil’s healthcare system, especially among older adults [2].

In February 2020, the World Health Organisation forecasted a 60% increase in cancer cases worldwide over the next two decades, particularly in LMIC [3]. The differences in cancer mortality between HIC and LMIC are striking, mostly due to insufficient preventive measures and diagnosis of cancer in advanced stages [4]. In Latin American countries and the Iberian Peninsula, healthcare systems are not prepared to support older patients with cancer, mainly due to a lack of resources, training and qualification of health providers in geriatrics [5].

These current and future changes in the incidence and prevalence of cancer make it necessary to understand how care for older individuals is delivered in such large and diverse countries. The implementation of a geriatric oncology service is challenging in both HIC and LMIC, as there is a significant demand for economic and human resources needed for structure and training [6]. In Brazil, despite its limited budget, the public health system is considered a model for several other countries owing to its organisational structure [7].

The Octavio Frias de Oliveira Institute of Cancer of Sao Paulo (ICESP) is part of the tertiary healthcare public complex at the Clinics Hospital of the University of Sao Paulo Medical School (HCFMUSP). It is considered a model of excellence in oncological care and is one of the largest cancer treatment centres in Latin America [8]. The ICESP provides care to 72,000 patients per year, among whom 54% are aged 60 years or over [9]. It has 500 beds, 85 of which are dedicated to intensive care. Each month, 6,000 patients diagnosed with cancer are treated in the institute, and more than 25,000 outpatient medical consultations, 250,000 exams, 7,000 radiotherapy sessions, 4,500 chemotherapy sessions and 600 oncological surgeries are carried out [10].

The ICESP’s geriatric oncology service was created in 2011 to improve the care of older patients. At that time, two geriatricians began outpatient care activities, assessment of hospitalised patients and surgical risk assessment for patients aged 80 years or older, and started to hold weekly scientific meetings. Medical residents from the HCFMUSP geriatrics service also became part of the team with periodic rotations. Since then, the demand for geriatric support has grown exponentially, with more than 80 requests for outpatient assessments per month. In 2017, motivated by an increase in demand, a therapeutic decision (TD)-making outpatient clinic was created to prioritise care for patients who were about to start cancer treatment. Other important events during this time included the hiring of a third geriatrician and the founding of a fellowship programme in geriatric oncology.

The objective of this manuscript is to describe the years of the geriatric oncology service at the ICESP and the challenges involved in implementing this programme in the public healthcare system of an LMIC country.

## Methods

This study uses a narrative descriptive design. We conducted a general retrospective descriptive analysis of data collected from the routine structured medical records of patients referred to the ICESP geriatric oncology service from 2011 to 2021. This study was approved by the institutional ethics committee. We analysed data that had been collected during clinical evaluation from the beginning of the programme, and which were managed securely using the Research Electronic Data Capture (REDCap) platform. All patients aged 60 years or older who had undergone at least one inpatient or outpatient assessment by the geriatric oncology service in the last 10 years were included in this report. Patients who did not have geriatric assessment (GA) data recorded in REDCap were excluded.

The variables included in the descriptive analysis were those extracted from the GA at the ICESP, and were based on validated scales and sociodemographic data, as described in Table 1. Each outpatient clinic has a specific protocol, with some differences in the scales used to assess each domain. Table 1 presents a compilation of all of the scales and their respective domains, as well as the cutoffs at which they are considered impaired. We describe our results using data retrieved from an electronic database according to age, sex, literacy level and frailty status. Continuous variables are described as means and SD, and categorical variables as counts and percentages.

## Results

### Composition of and activities performed by the geriatric oncology service at the ICESP

**Figure 1.**
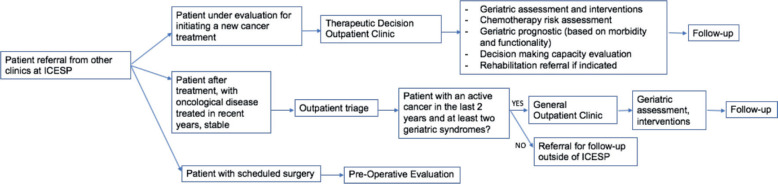
Flowchart of the outpatient geriatric oncology service at ICESP.

**Figure 2.**
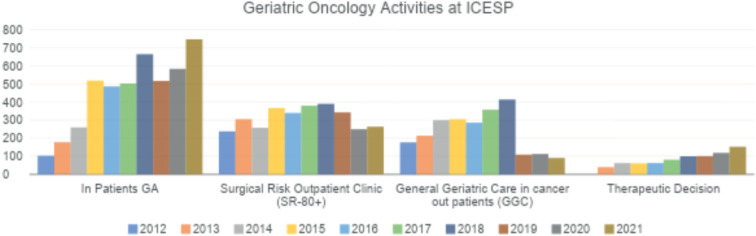
Number of consultations performed by the geriatric oncology clinic per year from 2012 to 2021.

Currently, the geriatric oncology service comprises three staff assistant geriatricians with a workload of 20 hours per week and three volunteer geriatricians with a workload of 10 hours per week. Older patients enrolled at the ICESP were referred to a particular type of outpatient care as appropriate for their needs, each of which has a specific protocol (Figure 1). Each type of outpatient care uses different scales to assess each domain to better meet the clinical demand. The geriatric oncology service is evaluated by the geriatrics team with the collaboration of medical residents from the HCFMUSP geriatrics service. The current allocation of patients who have accepted geriatric care and the number of consultations performed at each clinic between 2012 and 2021 are shown in Figures 1 and 2, respectively.

### Description and general data of the activities performed in each ICESP geriatric oncology service clinic

#### Triage outpatient clinic

The triage outpatient clinic was designed for the initial assessment of patients referred for GA by other ICESP clinics, mainly oncology and surgery specialties. At this clinic, GA evaluates whether a patient meets the criteria for follow-up by the geriatric oncology service. It is important to emphasise that this outpatient clinic was recently created to select patients who would benefit the most from geriatric care, as there has been a growing demand in this area. Data from 310 patients referred to the triage outpatient clinic between 2019 and 2022 were retrieved. The mean age was 77.9 (SD 10.9) years and the two main reasons for referral were multimorbidity (25%) and cognitive decline (24%). Only half of these patients had active cancer, of which the most common were gastrointestinal (20.9%) and prostate (22.9%). Table 2 shows the general characteristics of the patients assessed in this study.

#### General geriatric care outpatient clinic (GGC)

The inclusion criteria for follow-up at this clinic are being aged 60 years or older, having a confirmed cancer diagnosis (active disease or undergoing cancer treatment), and being listed under at least two impaired domains in the geriatric evaluation (Table 2). Accepted patients undergo an initial GA for the identification of comorbidities and geriatric syndromes (neuropsychiatric and/or neurodegenerative disorders; loss of at least two basic ADL; two or more falls in the past 12 months; one fall with serious consequences, such as fracture, disability and institutionalisation; and three or more chronic complications of diseases beyond cancer). It is important to point out that from 2019, there was a reduction in the number of appointments available so that other clinics could be expanded, especially the TD clinic. For this reason and also as a result of the COVID pandemic social isolation, there is a marked reduction in the patients of the GGC.

The mean age of patients at GGC was 78.7 (7.91) years. Table 2 shows the general characteristics of patients evaluated at the GGC. Among the patients referred for geriatric follow-up, the majority underwent surgical treatment (57%) and a minor portion received chemotherapy (18,8%) or radiotherapy (8%). Patients were discharged and referred to a general clinic outside the ICESP when there was no evidence of oncological disease or when referred for follow-up by the ICESP’s palliative care service.

#### Surgical risk outpatient clinic (SR-80+)

All patients aged 80 years or older with an indication for surgical treatment are referred to the geriatric oncology service’s surgical risk outpatient clinic in accordance with institutional protocol. The mean age of patients assessed from 2012 to 2021 was 83.9 (SD 4.95) years. The clinical assessment of surgical risk stratification is complemented by GA; thus, in addition to the usual surgical risk factors, cognitive, frailty criteria, and nutritional and delirium risks are evaluated. After assessment, patients are referred to the anaesthesiology department and undergo post-consultation with the nursing team. Figure 2 shows the number of patients assessed in this clinic, and Table 2 describes the clinical characteristics of the patients.

#### TD-making outpatient clinic

Patients aged 60 years or over are assessed in this clinic when referred from different oncology teams to obtain geriatric opinions to assist in TD-making. GA is performed to assist in the therapeutic planning of patients with a recent diagnosis of cancer or oncological disease progression. From 2012 to 2021, 468 patients were evaluated, with a mean age of 79.4 (SD 7.38) years. The proposed treatment options include one or more oncological treatment modalities. Among the patients referred for TD, the main initial treatment proposal was chemotherapy (52%), followed by surgical treatment (36%) and radiotherapy (12%). GA at the TD is standardised and based on validated scales and tests, as previously adopted elsewhere [20]. The protocol includes GA, sociodemographic and anthropometric data, specific characteristics of the cancer, assessment of performance status, and the use of prognostic scales. Clinical data and laboratory tests are evaluated to support the clinical management of older patients with cancer and estimate toxicity to systemic treatments and surgical risk. After evaluation at the TD, a report is issued with clinical diagnoses, geriatric syndromes, frailty criteria, toxicity risks and prognostic scores. The geriatric oncology team can propose medication adjustments, clinical compensation for comorbidities and referrals for nutritional therapy and physical rehabilitation before initiating cancer treatment. When geriatric evaluation detects limiting factors in the initially proposed treatment, adaptation or a change in treatment may be suggested. After the initial assessment, the patients are followed-up and undergo periodic GA approximately every 4 months. The final decision regarding the cancer treatment is made by the referring oncologist or surgeon.

### Geriatric inpatient evaluation

The geriatric oncology service does not have specific beds for hospitalisation and evaluates patients aged 60 years or older at the request of the oncology and surgery services. Between 2017 and 2021, 807 patients with a median age of 78 (SD 24.01) years were assessed. Most patients were admitted with acute conditions related to oncological diseases and treatment complications (*n* = 716, 88.7%). Almost 70% of the patients had active cancer (*n* = 554) and 39% (*n* = 217) had metastatic disease. Table 3 describes other clinical characteristics of the sample.

The most frequent demands for the service are perioperative evaluation and follow-up, assistance in the management of delirium and comorbidities, therapeutic planning and assistance with safe discharge. Patients are subject to clinical evaluation and GA; thus, a geriatric intervention plan can be formulated and suggested to the oncology and surgical teams. Follow-ups and frequency of reassessment are determined according to the patient's clinical status and reason for the inpatient evaluation request. Before hospital discharge, the geriatric oncology service advises the requesting team to refer patients for follow-up at a general geriatric outpatient clinic or to an outpatient primary attention health care service.

## Discussion

In this paper, we described the 10 years of activities at the ICESP's geriatric oncology service, highlighting the different settings in which it operates (outpatient, preoperative and hospital follow-up). We also described the profiles of the evaluated patients. There is virtually no literature on the comprehensive profiles of older Brazilian adults with cancer. This discussion focuses on the main challenges faced in implementing this program in the public healthcare system of an LMIC.

### Challenges of assistance

The percentage of older adults (aged 60 and above) among cancer patients at the ICESP is approximately 55%, similar to the worldwide incidence, in HIC and LMIC, such as the USA (54%), Australia (58%), France (62%), India (56%) and Mexico (43%) [5, 22–24]. Current cancer incidence and mortality rates in older adults are still higher in HIC, however, the absolute number of new cases and deaths in developing regions such as China, Southeast Asia, India and Latin America is proportionately larger [25, 26]. Regarding this high demand and the need to optimise resources, this report shows that patients referred for evaluation at the geriatric oncology service at ICESP are notoriously older (mean age above 77 years old), those with greater vulnerabilities, frailty and in need of multidisciplinary interventions.

Evidence supporting GA demonstrates that it has an important capacity to perform new diagnoses not captured by traditional oncological evaluation, including geriatric syndromes [27–29]. The present data show that the outpatients evaluated by the geriatric oncology service at the ICESP have a high incidence of the following GA domains impairments: 39%–52% had polypharmacy, 37%–45% had at least one impaired ADL, 57%–82% had at least one impaired IADL, 20%–84% had risk of malnutrition and 50%–73% have altered cognitive screening. Russo *et al* [30] described a similar experience in a French geriatric oncology centre, where of the 266 patients evaluated, a significant percentage had vulnerabilities identified in the GA: 47% were malnourished, 48% had compromised mood and/or cognitive function and 53% had a functional impact. A recent description of an Indian sample also indicated similar data: malnutrition (65%), cognitive impairment (18%) and polypharmacy (>50%) [23]. These data corroborate the need for GA to complement oncological evaluation so that these syndromes are not neglected and can be properly addressed.

A major obstacle identified, as described in the literature, is the unavailability of geriatricians and the lack of financial resources compared to the high number of older patients with cancer [31]. Population aging and the exponential increase in cancer rates make it difficult to refer older patients with cancer to geriatricians, even in large centres. Therefore, it is essential to create strategies to meet the demands of a hospital where the majority of patients are older and to understand which strategies have the most impact in terms of survival and quality of care [30].

The provision of assistance must be adjusted according to the local reality and team size, prioritising the most vulnerable patients. One feasible and cost-effective approach is to include nursing and allied health staff in the care of older cancer patients, especially in geriatric screening. As an established recommendation, it is important to provide specialised training and continuing education [32]. At the ICESP, regarding the high demand, the limited number of consultations and the lack of nursing staff available for geriatric screening, establishing strict referral criteria is essential for the organisation and planning of care. In our experience, promptly referring newly diagnosed patients to a TD clinic has enabled geriatric support that is better suited to the patients’ needs throughout the cancer treatment. Conversely, patients who do not undergo oncological treatment often wait longer for geriatric consultations.

At the ICESP, the institutional protocol requires that all patients aged 80 years and older undergoing surgery are evaluated by geriatricians. It is important to highlight that almost 50% of the assessments were for bladder cancer and non-melanoma skin cancer (which are highly prevalent tumors in this age group that have proposals for low-risk surgical intervention). Comparing the group of patients at SR- 80+ and other outpatient clinics (triage, CCG and TD), despite the slightly higher average age of this group (83.8 versus 77.9, 79.9 and 79.4 years old), a better performance in the geriatric domains is observed, due to a possible intuitive screening by the surgical teams. Other interesting points of our sample are in consideration of prostate and breast cancer. Prostate cancer surgeries, despite representing 10% of the cases, are performed for transurethral resection and orchiectomy rather than curative prostatectomy. Regarding breast cancer, a point to be discussed is the low incidence of surgeries, which can be justified by the fact that these patients are over 80 years of age, and most of the time, conservative treatments such as hormone therapy are offered.

A significant percentage of our patients had positive cognitive screening test results (even after adjusting for years of formal education) which does not necessarily reflect a diagnosis of clinically relevant cognitive impairment. In Brazil, the education level of the population remains low, and 30% of illiterate individuals are older adults. The older Brazilian population also has 3.3 years less than the average amount of schooling [33]. Better cognitive reserve can diminish cognitive losses in many age-related central nervous system diseases, and low cognitive reserve is a risk factor for them as well as for cancer-related cognitive impairment [34].

Nevertheless, it is possible that our patients were more prone to cognitive impairment due to their sociodemographic characteristics. This highlights the need for careful cognitive assessment to better tailor treatment plans and evaluate the decision-making capacity of older patients with cancer. Moreover, patients with lower cognitive performance should be addressed for associated conditions such as sleep disorders, fatigue, psychological distress, anxiety, depression and other ageing-related diseases and conditions (e.g., diabetes, cardiovascular disease, thyroid disease and frailty) [34].

### Challenges with multidisciplinary teams

Like most geriatric oncology services around the world, the ICESP's geriatric oncology service uses an assessment model based on GA, providing recommendations to assist clinics regarding individualised treatment and promoting geriatric interventions with an impact on health outcomes.

As an extra challenge, at the beginning of our activities, we had to demonstrate the role of geriatrics in the management of older people with cancer in a scenario where medical conduct was predominantly decided exclusively by oncologists, radiotherapists and surgeons. The growing number of patients evaluated in outpatient TD-making clinics reflects the recognition of the benefits and reliability of geriatric evaluations in decision-making in our practice.

One remarkable consideration is that, although there is a multidisciplinary team available for referral, which includes physiotherapists, speech therapists, social workers, nutritionists and nurses, there is still no interdisciplinary case discussion due to practical difficulties (appointments in different locations) and logistics (high demand for appointments). Therefore, interdisciplinary case discussion is a challenge, and we hope to implement it in the coming years.

The current geriatric oncology scenario in Brazil is similar to that reported in the international literature: there is a lack of adhesion of oncologists to the performance of geriatric evaluation or the application of screening tools [34–36].

A recent Australian study, which conducted an online interview with 93 oncologists to determine the factors influencing the prescription of chemotherapy for older patients, showed that only 5% of the respondents routinely used GA, and only 14% used any screening tool [37]. In the same study, when asked about the evaluation of cognitive, functional, nutritional and psychological status, most physicians reported evaluating these domains informally, according to clinical judgment, rather than using a validated tool. Possible reasons for the low use of geriatric tools by oncologists were lack of familiarity, doubt regarding the benefit of patient care, lack of time and confidence in clinical perception, and care provided to the patient.

The International Society of Geriatric Oncology, the American society of clinical oncology and the European Organisation for Research and Treatment of Cancer recommend a routine, structured GA for cancer patients aged ≥65 years for whom chemotherapy is considered [32]. Despite the potential benefits of GA and its recommendation in the main international guidelines for oncology, the best model for its implementation remains uncertain and varies greatly in different scenarios.

### Social support challenges

At the ICESP, older patients' social issues are a daily challenge. The literature presents some instruments to directly evaluate tangible social support, such as the medical outcomes survey social support survey [38]; however, for practical reasons, it was not possible to include this in our protocol. However, social evaluation is considered a key issue, and our team relies on the essential participation of social workers to help us overcome these barriers. In this context, the assessment of family support is essential. The significant impairment of IADLs even in the outpatients group, indicates a greater demand for care, a probably greater burden for informal caregivers, higher expenses with transport and structure for care and eventual absence in medical appointments. Issues like these need to be identified, addressed and inserted in the discussion of the global care plan. Limited social support remains one of our biggest challenges, and it requires comprehensive and personalised care to address individual needs.

## Conclusion

Quality assistance and training of health professionals is our priority as an acknowledged university oncology centre in Latin America. In the past 10 years, our geriatric oncology team has adjusted its characteristics to meet hospital demands, and to raise awareness among the oncology teams about the benefits of using GA and promoting multidisciplinary discussions. However, limited social support remains a major challenge. Future research should focus on improving scientific production while maintaining the best care and assistance for patients and while training physicians.

## Conflicts of interest

The authors declare that there is no potential conflict of interest to disclose.

## Funding

No funding was received for this article.

## Figures and Tables

**Table 1.** Domain’s variables used in the GA evaluation. *The cut-off value for educational groups was: 20 for illiterates; 25 for 1 to 4 years; 26.5 for 5 to 8 years; 28 for 9 to 11 years and 29 for higher levels [11]. #Score has to be adjusted to educational: for illiterates sum of two points, 1–3 years sum of one point (final score max of ten points) [12].

Domain	Measure	Description	Range of score	Adopted standard
**Functional status**	Activities of daily living (ADL) (Katz Scale) [10]	Measure performance basic activities of living, including self-care ability.	0–6 (higher score: better physical function)	**Normal:** score > 4 **Impaired:** score < 4
	Instrumental ADL (Lawton Scale) [11]	Measure ability to maintain independence in the community, manage the environment.	3–27 (higher score: better physical function)	**Normal:** score >25 **Impaired:** <25
**Cognition status**	Mini Mental State Examination [12]	Screening test to detect cognitive impairment based on assessment of cognitive functions.	0–30 (higher score: better cognitive function)	The cut-off value is based on educational level*
	10-point cognitive screener [13]	Brief screening test to detect cognitive impairment based on assessment of cognitive functions.	0–10 (higher score: better cognitive function)	**Normal:** score > 8 **Impaired:** score < 8 Score adjusted to educational level^#^
**Psychological state**	15-item geriatric depression scale (GDS-15) [14]	15-item self-report measure designed to assess and screen depressive symptoms among older adults.	0–15 (higher score: more depressive symptoms)	**Normal:** score 0–4 **Impaired:** score > 5
	4-item GDS-4 [15]	4-item self-report measure designed to assess and screen depressive symptoms among older adults.	0–4 (higher score: more depressive symptoms)	**Normal:** score 0–1 **Impaired:** score > 2
	Diagnostic and statistical manual of mental disorders 5th edition [16]	It classifies major depressive disorder based on ≥5 symptoms during a 2-week period		**Impaired:** Presence of major depressive disorder criteria
**Comorbidity**	Charlson age-comorbidity index [17]	It considers age and number/severity of comorbidities, calculating the risk of mortality in 1 year	0–37 (higher score: higher mortality). Each decade of age over 40 adds 1 point	**Impaired:** score > 5
**Nutritional status**	Mini nutritional assessment short-form [18]	Identity people with undernutrition.	Normal: >12 Risk of malnutrition: <12	**Impaired:** score < 12
	Body mass index	It is defined as the body mass divided by the square of the body and height, and is expressed in units of kg/m^2^		**Impaired:** IMC < 22 kg/m^2^
**Frailty**	Fried criteria [19]	It assesses physical frailty through five criteria: unintentional weight loss; weakness or poor handgrip strength; self-reported exhaustion; slow walking speed; and low physical activity	Non-frail: 0 Pre-frailty: 1–2 Frailty: >3	**Impaired:** score > 3
	Study of osteoporotic fracture criteria [20]	Another form to assess frailty, based on weight loss, sit-to-stand chair capacity and reduce energy level.	Non-frail: 0 Pre-frailty: 1 Frailty: >2	**Impaired:** score > 2
	Rockwood classification [21]	It provides a summary tool for clinicians to assess frailty and fitness based on their clinical evaluation		**Impaired:** score > 5
**Falls**	No. of falls in last 12 months	Indicates the number of times fallen in the last 12 months		**Normal:** None Impaired: >1 falls

**Table 2.** Comparison between oncogeriatric outpatient services at ICESP over time.

	Triage (2019 –2022)	GGC (2012–2019)	TD (2012–2021)	SR 80+ (2013–2019)
	*N* = 310	*N* = 967	*N* = 468	*N* = 1,700
Mean age (years, SD)	77.9 (10.9)	79.95 (10.9)	79.4 (7.38)	83.8 (4.95)
Gender Female Male	143 (46) 167 (54)	560 (58) 407 (42)	278 (59) 190 (41)	807 (47) 893 (53)
Type of cancer *n* (%) Breast Lung Prostate Urothelial Gastrointestinal Gynecological Head and neck Melanoma Skin non-melanoma Hematologic Other	35 (12) 7 (2) 71 (23) 3 (<0.1) 65 (21) 7 (2) 23 (8) 7 (2) 14 (5) 7 (2) 71 (23)	215 (22) 47 (5) 104 (11) 45 (5) 303 (32) 39 (4) 43 (4) 8 (<0.1) 39 (4) 50 (5) 74 (8)	88 (19) 21 (5) 15 (3) 11 (2) 252 (54) 10 (2) 16 (4) 1 (<0.1) 20 (4) 15 (3) 19 (4)	138 (8) 20 (1) 168 (10) 365 (21) 233 (14) 76 (4) 154 (9) 54 (3) 433 (26) 0 (0) 59 (4)
ECOG *n* (%) 0 1 2 3 4 Missing data	-	75 (8) 266 (28) 294 (30) 147 (15) 61 (6) 124 (13)	34 (7) 137 (29) 157 (34) 110 (24) 28 (6) 2 (<0.1)	204 (12) 697 (41) 425 (25) 204 (12) 68 (4) 102 (6)
Polypharmacy *n* (%)	146 (47)	499 (52)	204 (44)	667 (39)
Impaired ADL *n* (%)	120 (39)	435 (45)	181 (39)	629 (37)
Impaired IADL *n* (%)	-	737 (76)	385 (82)	969 (57)
Nutritional risk *n* (%)	-	270 (28)	391 (84)	340 (20)
Impaired cognition screening *n* (%)	156 (50)	-	342 (73)	1082 (64)
Depressive symptoms *n* (%) Missing data	81 (26)	-	102 (22) 56 (12)	158 (9) -
Impaired G8 screening *n* (%)	159 (51)	-	-	-
ECOG: Eastern Cooperative Oncology Group; ADL: activities of daily living; IADL: instrumental activities of daily living; G8: geriatric-8 screening tool				

**Table 3.** Characteristics of the geriatric inpatient evaluation from 2017 to 2021 (*N* = 807).

Mean age (years, SD)	78 (24.01)
Gender Female Male	353 (44) 454 (56)
Type of cancer Breast Lung Prostate Urothelial Gastrointestinal Gynecological Head and neck Melanoma Skin non-melanoma Hematologic Other Missing	64 (8) 27 (3) 118 (15) 97 (12) 198 (25) 27 (3) 87 (11) 4 (0.5) 71 (9) 40 (5) 32 (4) 52 (6)
Polypharmacy	378 (47)
Impaired ADL Missing data	425 (53) 61 (8)
Nutritional risk	343 (43)
